# A Neutron Reflection Study of the Dissolution of Miscible Glassy Polymer Films over a Range of Temperature

**DOI:** 10.1021/acs.macromol.5c02222

**Published:** 2025-10-14

**Authors:** Guangcui Yuan, Sushil K. Satija, Thomas R. Murray, Jack F. Douglas

**Affiliations:** † Center for Neutron Research, 10833 National Institute of Standards and Technology, Gaithersburg, Maryland 20899, United States; ‡ Department of Chemistry, Physics & Material Science, 3338 Fayetteville State University, Fayetteville, North Carolina 28301, United States; § Materials Science and Engineering Division, 10833 National Institute of Standards and Technology, Gaithersburg, Maryland 20899, United States

## Abstract

Although the mixing of miscible liquids is generally thought to be relatively well-described by classical Fickian diffusion models, it is well-known that the physics of glass-formation can greatly alter the dissolution dynamics of polymer materials brought into contact with solvents and other polymer materials. Despite the immense practical importance of this phenomenon in many contexts where polymers are “blended” (e.g., polymer recycling), models of the dissolution dynamics of glassy polymer materials tend to be highly phenomenological and to have limited general applicability. This situation is understandable given the limited fundamental understanding generally of glass formation, pointing to the need for high-resolution measurement methods on model materials and an appropriate theoretical framework to elucidate the fundamental nature of the polymer dissolution process under realistic physical conditions. In the present work, we restrict our consideration to the interfacial dynamics of a model system of this kind, composed of two thermodynamically miscible, but glassy, polymer films formed through spin-coating and film floating techniques. We utilize neutron reflectivity (NR) to characterize the interfacial mixing dynamics in these stacked glassy polymer films. The initial stage of the interfacial mixing is found to be somewhat similar to Fickian interdiffusion of miscible liquids, although we observe a fractional power-law growth of the interfacial width in this transient regime with an exponent near 1/3, as commonly observed in the coarsening of phase-separating polymer blends. After an induction time t* over which the interfacial concentration approaches that of the fully dispersed polymer mixture, we generally observe a transition to a regime in which the “mixed” polymer in the interfacial region between the films invades both films in a front-like fashion. We adapt phase field modeling of “solid” material dissolution to qualitatively understand and characterize this evidently complex non-Fickian dissolution process. The rates of the propagating composition wave, as well as the evolution of the interfacial width separating the relatively stable “uniformly mixed” polymer material from the relatively unstable unmixed polymer material, are quantified. Interestingly, this frontal dissolution process in glassy miscible polymer films closely resembles the phenomenology of Case II non-Fickian diffusion commonly observed in the dissolution of glassy polymers by solvents, and we suggest that the latter corresponds to the situation in which one of the layer components is not glassy so that the dissolution wave propagates only in one direction.

## Graphical abstract

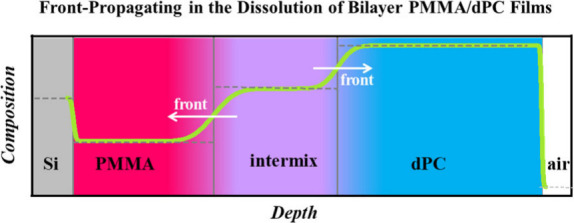

## Introduction

The dissolution of glassy polymers is a fundamental problem in diverse technological applications,^1–11^ but modeling of the dissolution process has been largely limited to the case of unentangled polymer materials at temperatures well above the glass transition temperature (*T*_g_), where classical Fickian diffusion models appear to describe the dissolution process rather well.^12,13^ While evidence supports this Fickian diffusion model at temperatures far above *T*_g_ of both polymer materials,^14^ the nature of the dissolution process is commonly observed to be quite different when the dissolving polymer material is in its “solid” glass state at temperatures below its *T*_g_. In particular, the dissolution process of glassy polymer films by a low-mass solvent is often found to resemble crystal melting in the sense that the dissolution process occurs in a wavelike rather than diffusive fashion in the variation of the local order and composition of the material, depending on the specific mechanism of melting.

Given the importance of this phenomenon in applications, an enormous literature has emerged related to the modeling of polymer dissolution.^1–11^ Despite all the modeling effort, we do not believe that any general predictive theoretical framework currently exists to fundamentally explain the rather distinctive fashion by which glassy polymer films dissolve. Apart from the many theoretical difficulties in understanding the physics of glass formation and polymer “entanglement”, we believe that part of the problem in developing a theoretical framework is due to the limited experimental information about the interfacial dynamics of the dissolution process on a molecular scale and over a long time scale in which the dissolution process occurs. We decided that neutron reflectivity (NR) measurements on carefully prepared glassy bilayer films could be instructive about the essential physical nature of the dissolution process. In particular, we examine the detailed development of the composition profile at the interface when a thin entangled polymer film of deuterated polycarbonate (dPC) is placed into direct contact with a thin unentangled polymer film of poly­(methyl methacrylate) (PMMA). Our choice of an entangled PC film in the measurements was made to enable the manipulation of the films without brittle fracture.

The present work is an extension of a previous study focused on the interfacial dynamics of model miscible bilayer polymer films made from hydrogenated and deuterated polystyrene,^15^ at temperatures well below the glass transition where the evolution of the interfacial region is limited to a nanoscale interfacial layer and the polymer mobility is sufficient to allow for slow “interfacial healing” rather than interdiffusion found in liquids. Notably, the broadening of the interfacial region was found to be highly non-Fickian, where the interfacial width seemed to approach a temperature-dependent constant thickness on the order of a nm at long times for the polymer materials deep in their glass state. In the general case, while interdiffusion processes are no doubt involved in compositional changes, the evolution of the composition in the interfacial region can be expected to depend on the states of the material. The term “interfacial healing” introduced by Yuan et al.^15^ is to describe the evolution in the composition of interfaces between different materials brought into contact, which applies even when the materials involved are not necessarily Fickian liquids. Our current NR measurements on dPC/PMMA films follow the interfacial dynamics at temperatures where sufficient mobility exists for the polymer films to mix on a reasonable time scale, but both polymers are in a temperature regime where they should exhibit glassy dynamics in the precise sense described below. Note that the transesterification reaction between two polymers, which does not occur readily without a catalyst and requires higher temperatures, should not be a concern in our study.

At the outset, we acknowledge that there have been numerous previous theoretical and experimental studies of the interfacial dynamics of polymers, and we mention some interesting review papers by Kausch and Tirrell,^16^ Klein,^17^ Stamm and Schubert,^18^ and Bucknall.^19^ In most of the systems studied quantitatively previously, the polymers are miscible, and the annealing temperature at which dissolution occurs is well above the *T*_g_ of both molecular species. In those cases, the interdiffusion behavior resembles Fickian diffusion, where the width of the concentration profile, or the distance the dissolving polymer has diffused, increases proportionally to the square root of time as *t* ^1/2^.^14,20^ Therefore, the dynamics of the interfacial broadening have been related at least qualitatively to the rate of molecular diffusion. Additionally, various experimental investigations in polymer–polymer interdiffusion have touched upon the non-Fickian interfacial dynamics in the literature.^21–26^ We note that there have also been many studies of immiscible polymer films^19,27–30^ where the degree of polymer immiscibility ultimately regulates the width of the interfacial composition profile at a long time scale to a spatial scale on the order of the correlation length for composition fluctuations.^31^ Since many polymers tend to be immiscible, the interfacial dynamics of immiscible films have practical importance in applications. Nonetheless, the physical situation in which polymer films dissolve when put in contact with a “good” solvent or with a miscible polymer film has numerous applications, and our work focused on the dissolution process in these systems. Our particular concern in the paper is in the detailed nature of the process in which the interface initially “heals” to some locally “equilibrated” state having a uniform composition, and then how this thermodynamically more stable interfacial region broadens to encompass the entire material at long times. As we anticipated, this broadening process occurs in a way highly distinctive from the interdiffusion of fluid mixtures.

We find that the interfacial broadening in our stacked films occurs in a fashion somewhat similar to the dissolution of nonpolymeric solid materials (e.g., minerals), and we utilize a phase field framework^32^ previously proposed for this type of dissolution process^33^ to semiempirically quantify the dissolution of our dPC/PMMA bilayer films. We mention that the solid-like dissolution process is also highly reminiscent of film melting, which has recently been claimed to be observed in ultrastable vapor-deposited glass films.^34–36^ Moreover, measurements have revealed the existence of a finite zero frequency shear modulus in model glassy materials in the vicinity of *T*_g_,^37–39^ so that it is plausible to model “glassy” polymer films as being in an equilibrium “solid” state. In line with this heuristic identification of the material state of glasses, the elastic properties of polymer films in their glass state are often modeled by assuming that these materials can be described as ideal Hookean elastic materials.^40,41^ At the same time, it should be appreciated that there is no evidence that materials in their glass state exhibit any overt thermodynamic phase transition at *T*_g_, so that the temperature at which glassy material should transform from a “solid” to a “simple liquid”, in the sense of a material exhibiting Fickian diffusion, is not presently clear. On the other hand, glass-forming liquids showing a sharp variation of relaxation times at elevated temperatures, so-called fragile-strong glass-forming liquids, have been observed in simulations of model metallic glass material to exhibit a sharp peak in their specific heat at well-defined temperatures well above the kinetically defined *T*_g_, which has been attributed to a rounded thermodynamic transition, and it has been suggested that this feature might arise in glass-forming liquids generally, although the thermodynamic transition is much less conspicuous in most glass-forming liquids where the glass-formation process is less cooperative in nature.^42^ This type of glass formation is observed in diverse glass-forming materials,^43^ but as with many aspects of glass-forming liquids, there is little scientific consensus on its physical origin. Angell^44^ has offered a possible general theoretical framework for understanding “ordering” processes more general than crystallization.

An interesting implication associated with the wavelike dissolution of glassy polymer materials (termed Case II diffusion in the engineering literature when one of the fluids is a small molecule solvent), while the undissolved polymer materials are in a glassy state, is that the dissolution process should, by consistency, transform to being Fickian at sufficiently high temperatures. While it is commonly stated in modeling studies of Case II diffusion that such a transition must occur as temperature is increased to a sufficiently high temperature at which polymer materials become “simple” fluids, so that ordinary diffusion describes the material dynamics.^1^ However, measurements actually indicating such a transition are sparse. There is some evidence for such a crossover from Case II to Case I or Fickian interdiffusion upon heating in the dissolution of a model polymer (PMMA) in its glass state by a low molecular mass miscible solvent (methanol),^45,46^ but the altered dynamics in the heated sample does not exactly conform to the expectations of Fickian interdiffusion, perhaps because of the inherently limited temperature range studied. There are further limited studies of this kind that suggest that a crossover to Fickian dynamics might occur, but we have found no study that shows any such crossover in a fully convincing way. Nonetheless, the limited evidence available from the various fragmentary studies indicate that Case II dissolution dynamics of glassy polymer materials seems to persist well above *T*_g_, which raises the question about what temperature or temperature range should this purported transition occur, or more specially, at what temperature does a polymer material behave as “simple” liquid with respect to its dissolution dynamics. In the engineering literature, it has long been observed that fluid dynamics in polymers appears to emerge at a specific “fluid temperature”,^47,48^ which is typically around 1.2 *T*_g_ in polymer materials. This temperature happens to also be close to a characteristic temperature at which the universal non-Arrhenius Vogel–Fulcher–Tammann^49–51^ temperature dependence of the structural relaxation time and diffusion coefficient of polymeric glass-forming liquids emerges.^52^ This unequivocal characteristic temperature in the dynamics of polymer liquids^53^ has a long history in polymer science,^47,54,55^ where in the context of processing applications it is designated the “liquid–liquid transition temperature” *T*_ll_. Unfortunately, there is little general agreement in the polymer science community regarding the fundamental physical interpretation of this apparent transition temperature from a “simple” fluid to a fluid exhibiting “glass dynamics”. Dudowicz et al.^52,56^ suggested that this crossover temperature corresponds to the characteristic temperature *T*_c_ of glass-forming liquids, a widely tabulated property in simulations and experiments on glass-forming liquids.^57,58^ Now, if we adopt the working hypothesis that this onset temperature for simple fluid dynamics in our film dissolution measurements should occur at *T*_c_, then we may expect Fickian diffusion to occur far above *T*_g_. For example, the lowest *T*_g_ of the polymers that we study here is around 88 °C and an estimated onset temperature for glassy dynamics around 1.2 *T*_g_ (a typical *T*_c_ value for polymer materials)^52,56,57^ would imply that we should expect a transition to Fickian dynamics for this polymer to occur around 160 °C. It is evident that non-Fickian interdiffusion dynamics should be the *norm* for mutual dissolution of miscible polymers at moderate processing-relevant temperatures, since many polymers start to thermally degrade before reaching the temperature range in which Fickian interdiffusion provides an adequate description of the polymer mixing process. On the other hand, the chance for seeing the transition between Case II to Case I dissolution should be much favorable when one of the components is a low mass solvent having a low *T*_g_, and indeed the limited evidence of a crossover in the dissolution dynamics from Case II to Case I mixing dynamics has only been reported in this type of system. We note that a transition from Case II to Case I mutual dissolution and near Arrhenius dynamics has been clearly documented in the case of a miscible polymer blend of PMMA and styrene-*co*-acrylonitrile copolymer under a processing condition comparable or higher than 1.2 *T*_g_ where the polymer components have a rather typical *T*_g_ around 100 °C.^14^ Based on these arguments and observations, we would not expect to see any transition to Fickian interdiffusion in our stacked glassy polymer films upon heating over a wide temperature range above the estimated *T*_g_ and 1.2 *T*_g_ range, and this expectation seems to be confirmed in the NR measurements described below.

In the present work, we provide a NR investigation into the evolution of concentration profiles between bilayer polymer films. Over a range of annealing temperatures (130 to 145 °C), the overall films are in a glassy state in the precise sense indicated above. The interface between the films has a relatively high mobility. Meanwhile, the polymer materials are also miscible under equilibrium thermodynamic conditions, so that there is a tendency for the polymers to “mix”, to form a material of uniform composition, except in the vicinity of the film boundaries where preferential polymer interaction leads to persistent deviations from compositional uniformity. We quantify how the interfacial profiles evolve over time in this type of bilayer material with unprecedented resolution. We also assessed the qualitative effect of varying temperatures on the interfacial dynamics. The evolution of the concentration in the bilayer film toward its uniform mixed composition state is found to occur in two distinct states. There is an induction process during which the interfacial region between two pure polymer films undergoes a non-Fickian evolution, leading to an intermediate layer with a composition that is apparently comparable to the uniformly mixed composition at long times (*Φ*_∞_). After an induction time (t*) for this compositional relaxation in the interfacial region between the films, we observe that the thickness of this intermediate mixed region increases as propagating fronts extend into both pure films. In this intermixed region, the composition transitions from that of the pure film to a mixed state *Φ*_∞_, where the rate of each front differs. This difference in the front propagating rate in each direction presumably reflects the asymmetry between the mobilities in the two pure polymers. Given the absence of an established theoretical framework to describe this phenomenon or the process of glass formation, we have introduced a new descriptive term to describe our observations. The films are said to undergo “mutual dissolution” of each other, and we describe the dissolution process as “frontal dissolution” as the evolution of the interfacial region involves the movement of two composition profiles fronts having pulse-like shapes, i.e., “fronts”. Some evidence is given that the temperature dependence of this remarkable collective intermixing process also reflects the positive cooperative diffusion coefficient (*D*_c_) governing the regression of macroscopic composition fluctuations in miscible materials. The phase field model of the dissolution of a solid material by a liquid provides some qualitative insight into the front process that we observe, the dual propagating fronts emitted from the quasi-equilibrium intermix region having a relaxed composition *Φ*_∞_. The interfacial width of the propagating fronts is found to take a nearly constant value on the order of a few nm and appears to be remarkably insensitive to temperature, similar to the expected trend for the static correlation length (*ξ*_o_) of a polymer blend in the one-phase miscibility region far from the critical point for phase separation.

## Experimental Section

### Sample Preparation

The dPC was synthesized following a procedure reported by Yoon et al.^59^ using phenol and acetone-d6 as starting materials. The molecular mass and the polydispersity of the final dPC used in this study are *M*_n_ = 8.4 × 10^4^ g/mol and *M*_w_/*M*_n_ = 2.45, respectively, determined by gel permeation chromatography (based on a polystyrene reference standard) with N, N-dimethylformamide as the solvent. *M*_n_ and *M*_w_ denote the number and weight-averaged molecular mass, respectively. The entanglement molecular mass (*M*_e_) for bisphenol A polycarbonate is 1660 g/mol, which is equivalent to 6.5 repeating units. The theoretical estimate of the radius of gyration (*R*_g_) for dPC is 11.0 nm, and the entanglement spacing is around 3.79 nm.^60^ The molecular mass of PMMA used in this study is *M*_n_ = 4000 g/mol (Sigma-Aldrich, Inc.) with a polydispersity *M*_w_/*M*_n_ of 1.06, which is well below its *M*_e_ (approximately 12500 g/mol). The theoretically calculated *R*_g_ for this PMMA is 1.6 nm.^60^ The *T*_g_s of dPC and PMMA are 156 °C and 88 °C, respectively, measured by differential scanning calorimetry (Mettle Toledo DSC822e) with a 10 °C/min heating rate for the second heating cycle. The heat flow vs temperature plots for pure components and blends presented in the SM (see Figure S1) demonstrate that the two components are miscible in any ratio.

The samples prepared for the NR experiment were dPC/PMMA bilayer films consisting of a dPC layer on top of a PMMA layer with polished silicon wafers as substrates (7.5 cm diameter, 0.5 cm thickness). The bilayer samples were prepared using spin-coating and floating techniques. Before spin coating, the silicon wafers were cleaned by treatment with freshly prepared “piranha” solution (70/30 v/v H_2_SO_4_ (50%)/ H_2_O_2_ (30%)) at 90 °C −100 °C for 45 min and then rinsed with a copious amount of distilled water and dried with a stream of technical grade nitrogen. Next, the silicon oxide layer was removed from the cleaned wafer by etching in a 5% HF solution. An approximately 45 nm thick PMMA layer was deposited on the wafer from the PMMA solution prepared in toluene by spin coating at 2500 rpm. The cast PMMA layer was annealed at 130 °C for 2 h under a vacuum to remove residual solvent and relax the stresses built during the spin coating process. The top dPC layer (approximately 70 nm thick) was prepared by spin coating a solution of dPC in 1, 1, 2, 2- tetrachloroethane on another clean silicon wafer. By immersing the wafer in distilled water, the dPC layer was floated off onto the water surface and then picked up on the wafer, which was spin-coated with PMMA in advance. Bilayer films were placed under vacuum at 90 °C for 24 h to remove residual solvent and water trapped between the layers before use. X-ray reflectivity (Bruker, D8-Ddvance) was used to characterize the single films with respect to film thickness and surface roughness.

Interfacial relaxation and the “mutual dissolution” of the glassy polymer films were accomplished by inserting the bilayer sample in a large, slotted aluminum block, preheated to the desired temperatures in a vacuum oven. The sample was centered inside the heating aluminum block enclosure with a small gap; thereby, heat transfer to the sample was maximized mainly through conduction. Hence, the time required for the sample to reach the desired temperature for the interdiffusion experiment was of no significant consequence. A thermometer inserted into the heating block monitored the sample temperature in the oven. The temperature was controlled to ± 0.3 °C. After the desired interdiffusion time, the specimens were quenched to room temperature by placing the substrate on a cool metal block. The time required to cool the specimen to a temperature below the *T*_g_ of PMMA, which will halt the interdiffusion process, was less than 20 s. All reflectivity experiments were performed at room temperature. Data at each temperature were taken from the same bilayer, which was tested about 10 to 20 times for different annealing durations, depending on the dissolution speed, until the dissolution process was completed.

### Reflectivity Measurement

The NR measurements were performed at the National Institute of Standards and Technology Center for Neutron Research (NCNR) using NG-7 horizontal reflectometer. The wavelength (*λ*) of the neutron used is 0.475 nm with Δλ/*λ ≈* 0.02. The reflectivity measurements were performed over a range of angles, and the data were presented as a function of the neutron momentum transfer perpendicular to the surface, *q* = (4π/λ)­sin*θ*, where *θ* is the incident angle of the neutron radiation. The angular divergence of the beam was varied through the reflectivity scan, and this provided a relative *q* resolution Δ*q*/*q* of 0.04. Since reflectivity observations are sensitive to the in-plane averaged neutron scattering length density (SLD) profile perpendicular to the sample surface, the SLD values were used to determine concentration profiles. The NR patterns are reduced with the Reductus program.^61^

### Refl1D Fitting

NR data was fit using Refl1D.^61^ In this program, a model SLD profile is proposed as a layered structure of material “slabs”. The fitting of models is completed using DREAM, a Markov chain Monte Carlo (MCMC) uncertainty analysis program. The DREAM methodology casts the fitting problem as determining the peak of the Bayesian probability distribution, with the probability of parameter set ***M*** given measured data ***D*** proportional to the probability of observing ***D*** given ***M***, scaled by prior information on the probability of ***M***, or P­(***M*|*D***) ∝P­(***D*|*M***)­P­(***M***). The Markov chain is formed by sampling a new point ***M*'** and accepting it with probability P­(***D*|*M*’**)­P­(***M*’**)/P­(***D*|*M***)­P­(***M***). With certain conditions on the selection of ***M*** '(detailed balance and ergodicity), the resulting chain will converge to a stationary distribution representing a sample from P­(***M*|*D***). Using this sample for Monte Carlo integration, we can estimate properties of this distribution, such as mean, variance, maximum likelihood, and credible intervals, and observe correlations between parameters. This method serves as a robust approach to sample multidimensional parameter spaces without selecting only a nearby local minimum (as can occur in gradient descent approaches), is able to identify multiple best fits when more than one solution is statistically feasible, and provides accurate uncertainty estimates for fitted parameters, as it explicitly preserves interparameter correlations. The quality of a model’s fit is determined by comparing it to the measured profile and calculating the *χ* ^2^, and the difference between fits is evaluated using Bayesian Information Criteria. The fitting of our NR data is described in more detail in the SM (see Figure S2).

## Results and Discussion

### Overview of Time-Dependent Composition Profiles

Figure 1 presents a selected set of reflectivity spectra, along with the evolution of composition profiles for a bilayer sample that was annealed at 135 °C. The modulation periods of the reflectivity spectrum (Figure 1a) change dramatically with time, indicating intense variation in layered structures as intermix proceeds. Correspondingly, the composition vs depth profiles can be visualized as a changing composite laminate or sandwich. Two distinct interfacial dynamics regimes can be identified: an “interfacial healing” regime (Figure 1b) in which the composition in the interfacial region approaches a value comparable to the fully mixed material and a “frontal dissolution” regime (Figure 1c) in which the unmixed parts of the films dissolve in a propagating manner. During an induction period t* over which interfacial healing occurs, the initially sharp interface between the as-cast films brought into contact in their glass state relaxes as the layers begin to interpenetrate through molecular diffusion mediated by intermolecular interactions between the polymers. After this initial local relaxation process at the film boundaries, the “frontal dissolution” process initiates. This process manifests as two composition fronts advancing in opposite directions, a phenomenon that extends the width of the interfacial region formed at short times in which the polymers are relatively uniformly mixed. As a result, a stable plateau in the composition develops that likewise propagates into both polymer films. This plateau scale is designated the intermediate mixing plateau length (*l*_mix_), and we quantify below the growth of this important length scale as annealing progresses. As noted before, the polymer composition within the plateau region between the mixing films remains nearly constant, apparently taking a value comparable to a uniformly mixed material. Throughout this mutual polymer film dissolution process, the thickness of the pure PMMA layer (*l*_PMMA_) and dPC layer (*l*_dPC_) progressively decreases. Ultimately, this process is exhausted over time as each film has almost fully dissolved in this manner. In Figure 1, this occurs after about 160 min. Note that the geometry confinement effect of the substrate makes it impossible to achieve a perfectly uniformly mixed state. As evident in the composition profile in Figure 1c, a PMMA-rich region remains adhered to the substrate even after 360 min of annealing.

**Figure 1:**
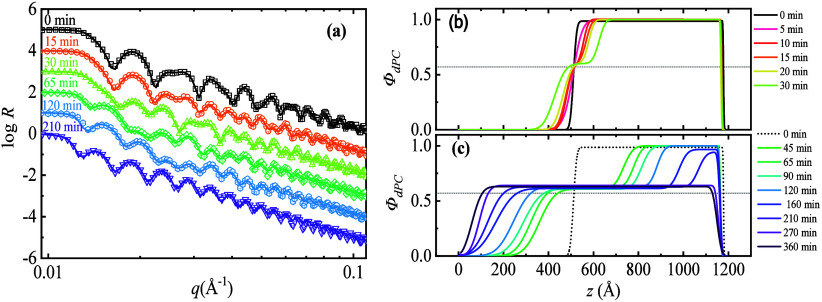
(a) Selected NR spectra for a dPC/PMMA bilayer sample annealed at 135 °C for various durations. The symbols are the experimental data, and each respective spectrum is offset by log *R* = 1 for clarity. The solid lines are the theoretical fits to the data from model SLD profiles, which are then converted into the composition profiles as shown in (b) and (c) (see SM: Figure S2). The horizontal dashed reference lines indicate theoretical *Φ*_*∞*_.

We want to clarify the role of substrate confinement and boundary interactions. Substrate confinement can influence the rate of interdiffusion, which depends on the interaction energy between the polymer segments and the substrate and the conformations of the chains.^29^ For PMMA near a Si substrate with an oxide surface which introduces attractive interaction, the effective range of the substrate on the interdiffusion dynamics is found to be between 3 *R*_g_ and 4 *R*_g_ of the PMMA.^29^ In our case, *R*_g_ for the PMMA used is 1.6 nm, while the thickness of the PMMA layer is around 45 nm. That is to say, the initial interface is about 30 *R*_g_ away from the substrate. In addition, the silicon oxide layer was purposely removed from the cleaned wafer by etching to reduce the attractive interaction. Therefore, the substrate effect should not be a concern for our discussion regarding the dynamics of “interfacial healing” and “frontal dissolution”, except that there is some residual nonuniformity of the polymer composition at the very late stage of mixing. Boundary interactions can also lead to variations in interfacial compositions due to the preferential segregation of polymers at the surface.^62^ However, in this study, we focused on the reverse process of phase separation: the interdiffusion of two miscible pure components across the interface. Consequently, no preferential segregation near the air surface was observed. Instead, the distortion of the composition profile near the air surface in the later stages of mixing is referred to as “front termination,” where the fronts have exhausted the materials in the films. Overall, we have taken necessary measures in experimental design to minimize the effects of substrate confinement and boundary interactions.

We admit that an error-function interfacial profile was initially assumed in our modeling of the above depth profiles, and we converted these profiles to the tanh profile fits given the occurrence of this functional form in phase field modeling. There may be other models that fit equally well, but the tanh model has some theoretical motivation, so we present our data in this form. We demonstrate in the SM (see Figure S2) that a hyperbolic tangent (tanh) profile closely resembles the error-function profile when accounting for corresponding interfacial width parameters. Considering experimental uncertainties, it is reasonable to conclude that the front profile can reasonably be described by either functional form or perhaps others. Properties such as the composition front velocity, profile shape, and position motivate the mode of fitting, however.

Additional SLD profiles for bilayer films annealed at other temperatures can be found in the SM (see Figures S3–S6). All fits for the annealed samples are checked with thickness and mass change compared to the corresponding as-cast films. The results indicate that the total mass of all components is conserved to within 1%, and the change in overall thickness is within 2%. The slight reduction of the overall thickness is commonly attributed to the residual solvent and trapped water, as it is typically found for floated bilayer films. It is observed that increasing temperature generally accelerates intermixing. At 145 °C, mixing is nearly completed within 45 min, while at 130 °C, the process is extremely sluggish, taking over 4 days. Furthermore, no penetration of PMMA into dPC was detected at 120 °C even after 24 h of annealing. We compare the measured uniformly mixed composition (*Φ*) with the theoretically calculated *Φ*_∞_ in the SM (Figure S7). The deviation of *Φ* from *Φ*_∞_ at all four temperatures is less than ±5%. The slight deviation is unavoidable due to two factors. First, the impact of diffusive interfaces has been overlooked in the calculation of *Φ*_∞_. Second, the preferential affinity of the polymer components for the boundaries of the films leads to compositional heterogeneity very near the boundaries in the long-time limit. Additionally, to highlight the differences between miscible and immiscible systems, we present the evolution of SLD profiles between an immiscible bilayer of dPC and a high molecular weight PMMA (*M*_n_ = 222000 g/mol) in the SM (see Figure S8). Due to the increased molecular weight of PMMA, these two polymers are immiscible, but some mobility is still observed in the films within the probed temperature range. The immiscible system does not maintain a uniform composition as it evolves into coexisting phases. This specific aspect, by gradually increasing the molecular weight of PMMA, will be discussed in more detail in a separate paper.

Before analyzing our NR observations on the interdiffusion process of dPC/PMMA films, we comment on previous observations and modeling of the dissolution of solid materials rather than liquid ones. In such systems, the solid material dissolves in a wavelike fashion,^31^ which can be modeled in a phase field perspective. The approach involves a model with a nonconserved order parameter associated with the solid material, along with a nonconserved field variable describing the local composition. In brief, the coupling of these field variables leads to a concentration front of the dissolving species that propagates at a constant velocity over a long time scale. Under steady-state growth conditions, both the shape of the frontal profile and the velocity of the moving front are constant. While there is no unique general exact solution that exists for this type of mean-field phase field, it is often found that a specific tanh-like profile provides a good description of the front shape, which reflects the local degree of ordering and local composition in the context of dissolution. We note that phase field theory has often been applied to more complex material states than crystalline materials, such as actin polymerization fronts, where the noncrystalline polymerized protein state is taken to be the “ordered” state.^63^ It is important to understand that, before the development of steady-state growth, there is usually an induction period during which the interfacial dynamics are somewhat similar to the interdiffusion of liquids; specifically, the interfacial width broadens according to a power-law over time.^64^ This early time interfacial broadening exponent is equal to 1/2 in mean-field theory; however, it is sometimes observed to take smaller values in cases where fluctuation effects in reaction-diffusion fronts become prevalent.^64,65^ Accordingly, we separately examine the short-time interfacial broadening that occurs during a transient induction period. In this phase, the local interfacial composition “heals” to a value comparable to *Φ*_∞_. The emergent local is presumably more thermodynamically favorable in our “dissolving” films. After the induction period, we observe the emergence of counter-composition fronts of dPC and PMMA that propagate in opposite directions as one polymer material invades the other. The situation we study here is contrasted with the case of glassy films being dissolved by a small-molecule solvent, where there is apparently only one front corresponding to the polymer material propagating into the solvent after the transient formation of a swollen interfacial layer. This distinct pattern of dissolution is likely due to high anisotropy between the mobilities of the solid polymer and the solvent. FTIR measurements have provided valuable insights into the role of solvent quality and polymer entanglement effects on this type of polymer dissolution process.^8–10,66^ Our mathematical analysis of the composition front in our films is similar to recent analyses for ordering fronts in self-assembled monolayers by near-edge X-ray absorption fine structure spectroscopy (NEXAFS)^64,67^ and to observations of crystallization in star polymer films.^68^

### Analysis of the Front Sharpness

The mutual dissolution process of the stacked polymer films occurs through the propagation of two fronts moving in opposite directions. The parameter *σ*_dPC_ refers to the interfacial width of the front advancing toward the pure dPC layer, while *σ*_PMMA_ refers to the interfacial width of the counter front advancing toward the pure PMMA layer. We analyze the evolution of the interfacial width as a function of time and examine the effects of temperature on this process. Considering the significant differences in the intermixing rates, which are in order of magnitude during annealing temperatures ranging from 130 to 145 °C, we first plot *σ* in Figure 2a as a function of *l*_mix_, which indicates the thickness of the growing region of “mixed” polymer. This parameter provides a measure of the progression of the film’s intermixing progress. Note that the interface profile is distorted at a late stage due to the exhaustion of material. Thus, only data before the exhaustion phase are shown. The observed common feature for both fronts is that the sharpness gradually increases and reaches a plateau. The interfacial broadening seems to be insensitive to temperature variation. The evolution of *σ* with diffusion depth for both fronts can be described by a stretched exponential function,

1 $$\sigma =\sigma_{\infty }\left\{1-\exp\left[-{\left(l_{\mathrm{mix}}/L\right)}^{\alpha }\right]\right\}$$

where *σ*_∞_ is the plateau value of *σ*, which describes the constant front sharpness during the steady stage, and *L* denotes a characteristic thickness of the intermixing zone for the process to enter the corresponding steady stage. This simple model, which was introduced in our earlier work on the interfacial healing of glassy hydrogenated and deuterated PS films well below their *T*_g_ ^15^ and in our later work on the initial interfacial broadening of entangled dPC and entangled PMMA films,^26^ was found to describe the interfacial broadening rather well. The exponent *α* is 1 in this case, probably related to the relatively higher mobility of the interface region.

**Figure 2:**
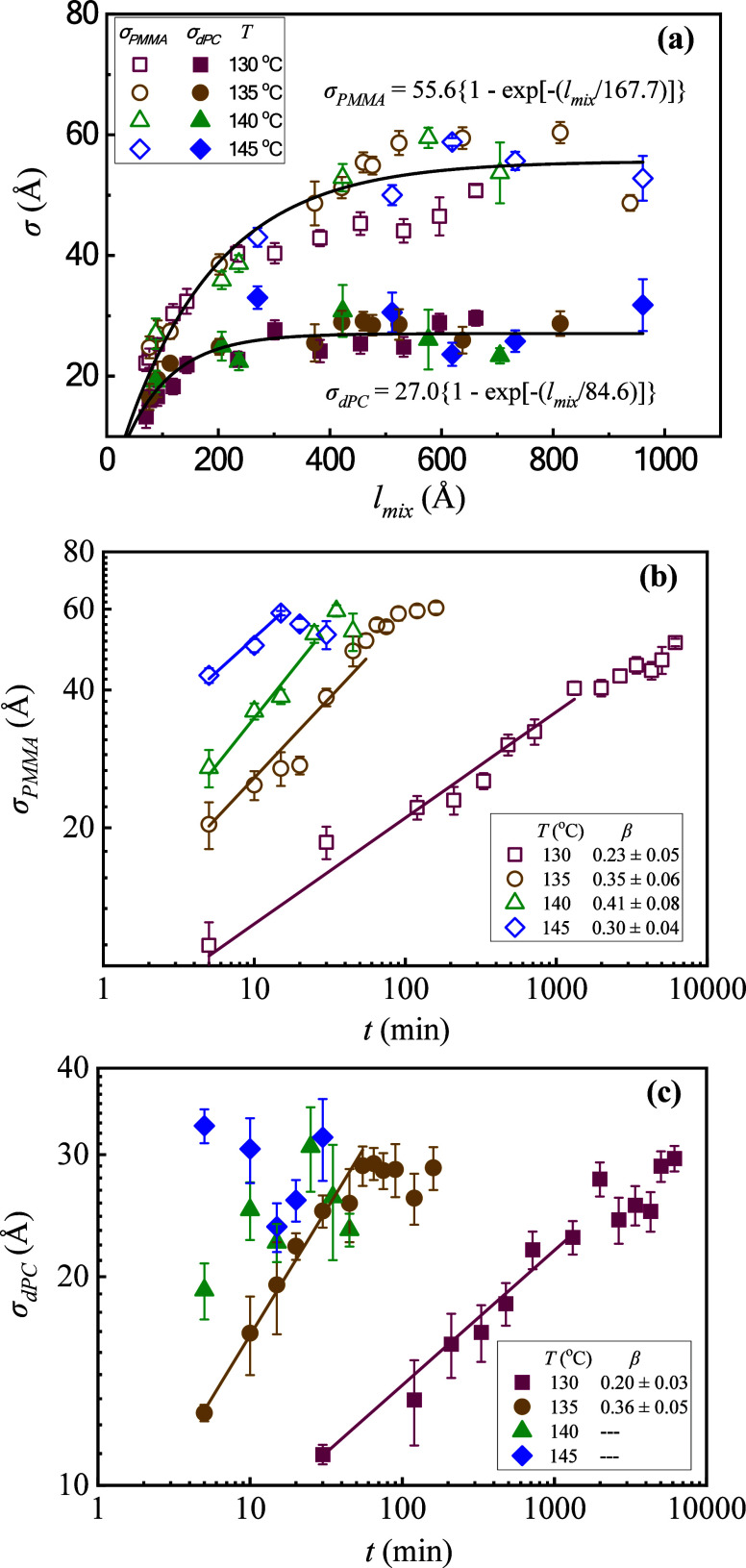
(a) The interfacial broadening of both PMMA and dPC advancing fronts as a function of intermixing depth *l*_mix_ for samples annealed at 4 temperatures. The solid lines represent the fitting results from the exponential functions. (b) The sharpness of PMMA front as a function of annealing time *t*. The solid lines represent the linear fit of log *σ* – log *t*, with slope provided in the inset. (c) The sharpness of dPC fronts as a function of annealing time *t*. The linear fit of log *σ* – log *t* for 140 and 145 °C was not conducted due to the very fast intermixing, resulting in a scarcity of data points for initial healing. The error bars represent the 95% credible intervals of DREAM fit.

One striking feature of the data shown in Figure 2a is that the scale of the plateau values *σ*_∞_ and *L* for PMMA and dPC differs by a factor of about 2 and shows a remarkable insensitivity to the variation of temperature. These trends were entirely unexpected. The plateau values *σ*_∞_ resemble typical static correlation lengths *ξ*_o_ of highly miscible bends,^69–71^ which are insensitive to temperature far from the critical point for phase separation. Distinct values of the correlation length have been reported in mixtures having a highly asymmetric structure,^31,72^ as in the present system. A very rough estimate of the correlation length far from the critical point *ξ*_o_would be the chain radius of gyration of the polymer, but in general *ξ*_o_ is predicted to depend rather sensitively on polymer geometry.^69^ A value in the range between a few nm and 100 nm can be expected in general. Another striking feature of the data shown in Figure 2a is that the characteristic length (thickness) scale *L* for the interface to develop a steady interfacial width parameter is about 3 times *σ*_∞_. At present, we cannot account for this striking phenomenology. Our observations above suggest that the intrinsic scale of composition fluctuation in the mixture at equilibrium in the one-phase region might set these scales rather than the mobility gradient near the film surface. This phenomenon obviously deserves further study.

The evolutions of the interfacial widths as a function of annealing time are shown in Figure 2b and Figure 2c for the PMMA front and the dPC front, respectively. During the induction period, the time dependence of the interfacial broadening is found to follow a power-law relationship, σ ∝*t* ^*β*^, where *β* is an interfacial roughening exponent. From the slope of the linear fit of the log *σ*-log *t* plot, *β* is determined. For interfaces of both fronts, we found *β* ≈ 1/3 at all annealing temperatures. This scaling exponent is quite reminiscent of the exponent frequently observed in the coarsening dynamics of phase-separating polymers and other fluid mixtures.^73,74^ Currently, it is too early to say whether the exponent 1/3 seen in the induction period or the “interfacial healing” period of interfacial dynamics observed in our measurements is instructive about the nonlinear diffusion processes generally in the classical spinodal decomposition theory. The relevance of the 1/3 exponent in our mutual diffusion measurements to the well-known 1/3 coarsening exponent in phase-separating blends needs to be examined on other polymer films undergoing mutual dissolution before any claims of general or specific theoretical relations can be made. However, we are confident that some type of nonlinear diffusion process is involved in the occurrence of this exponent in our measurements, as a coarsening exponent of similar magnitude has been observed in some other measurements on reaction diffusion. Note that, in our earlier study of interfacial healing, the interfacial width increases in this interfacial healing regime with a growth exponent of about 0.38, an exponent similar to observations on tumor growth^65^ and on the interfacial broadening of model self-assembled monolayers.^64^ This type of nontrivial exponent interfacial broadening is characteristic of fluctuation effects that are not normally incorporated in mean field models of reaction-diffusion fronts.^64,75^ At present, we cannot offer a simple rationalization of the nontrivial coarsening exponent observed in our polymer film dissolution measurements. A fundamental understanding of this exponent must await further experimental work and modeling of the mutual dissolution process.

### Quantification of the Front Propagation Velocity

Based on the observed trend, three stages are identified: the induction period, the propagation stage, and the exhaustion phase. An initial “interfacial composition relaxation process” occurs up to an induction time t* where front propagation initiates. Finally, there is “front termination,” where the fronts have exhausted the materials in the films. To view the progress of intermixing, the thickness change of each layer (i.e., the intermix zone Δ*l*_mix_, the pure PMMA Δ*l*_PMMA_, and the pure dPC Δ*l*_dPC_) is plotted as a function of *t*. An example is shown in Figure 3 regarding the intermix zone, Δ*l*_mix_ vs *t*. Linear fitting is conducted for the propagation stage, as shown by the straight solid lines in Figure 3a, and the slope is defined as the velocity of mixing *V*_mix_. Regarding the thickness change of pure PMMA layer and pure dPC layer, the Δ*l*_PMMA_ vs *t* plot and the Δ*l*_PMMA_ vs *t* plot, including the corresponding identification of *V*_PMMA_ and *V*_dPC_ at different annealing temperatures, can be found in the SM (see Figure S9). *V*_mix_ denotes the broadening rate of the intermixing zone, while *V*_PMMA_ and *V*_dPC_ describe the advancing velocity of the two fronts, which is also the consumption rate of the two pure components. The exhaustion phase is marked by the horizontal dotted lines, where the front propagation deviates from the linear growth and slows down dramatically. And the induction time t* is estimated from extrapolating the time at which the front position goes to zero.

**Figure 3:**
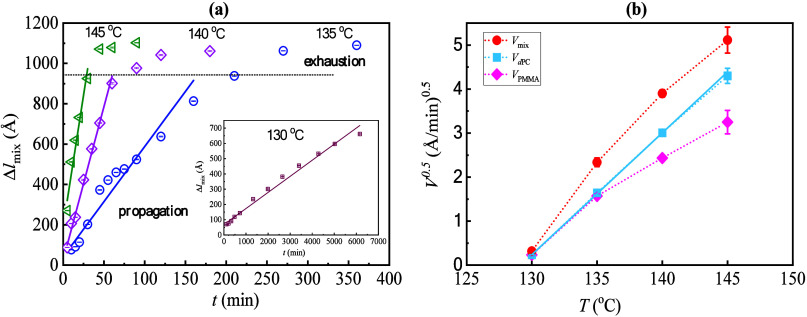
(a) Thickness expansion of the intermix zone as a function of annealing time (*t*) for bilayer samples annealing at 130, 135, 140 and 145 °C. The error bars show the 95% credible intervals of the DREAM fit, which are smaller than the size of the symbols. (b) Temperature dependence of the square root of the steady-state propagation velocities. Error bars come from the propagation of standard errors for the fit velocity parameters using least-squares regression theory. The solid line acts as a reference for the linear trend, allowing for visualization of the expected relationship between the variables.

The calculated values of *V*_mix_, *V*_PMMA_, and *V*_dPC_, along with their relationship to temperature (ln*V vs*. 1/*T*), are provided in the SM (see Table S2 and Figure S10). It is observed that the ln*V* vs 1/*T* clearly deviates from the linear Arrhenius relation, which is often reported in previous studies of frontal infusion of solvents into polymer materials near *T*_g_, known as Case II diffusion.^3,6,45,46,76^ The temperature dependence also differs from the strong Vogel–Fulcher–Tammann (VFT)^49–51^ ‘law’, which one might expect for the polymer translational diffusion coefficient and viscosity of polymer materials that are well above *T*_g_. Previous work on Case II diffusive transport has indicated a similar order of magnitude of the front velocity for a comparable temperature range, i.e., a change in front velocity on the order 10 for a 15 °C change in temperature in the glassy regime,^6^ except for the lowest temperature studied in the present work (i.e., 130 °C) where the front velocity drops precipitously. A linear plot of the square root of velocities versus temperature indicates that the front velocity seems to be extrapolating to zero as *T* approaches 129 °C, and the study of the front velocity becomes difficult at such low temperatures. The investigation of frontal growth in low-temperature conditions is complicated by a significant increase in the induction time that dictates the early stages of interfacial dynamics. The estimated induction times from Figure 3 are ≈ 0.7 min at 145 °C, ≈ 1.5 min at 140 °C, ≈ 7.5 min at 135 °C, and ≈ 650 min at 130 °C. As we mentioned earlier, there is no detectable frontal mixing between PMMA and dPC at 120 °C, even after 24 h of annealing. In previous studies of the interfacial dynamics of miscible glassy polymer by NR at temperatures appreciably below *T*_g_, we only observed the early stage of interfacial healing in which interfacial width coarsened in a non-Fickian fashion and was then observed to pin to a scale on the order of a nm at long times so that the interfacial dynamics could be described as “interfacial healing” rather than “interdiffusion”.^15^ The scale of the interfacial healing length was found to be consistent with the surface mobility gradient inferred from other measurements,^77^ and from simulation studies of polymer^78^ and other glassy films^79^ at low temperatures. The apparent arrest of the frontal mixing of the films at sufficiently low temperatures is thus consistent with previous experience.

Given the absence of any generally accepted predictive theory of Case II diffusion dynamics, our model of frontal mixing of the miscible films provides some insight into the dynamics of this type of mixing process. This model describes the transformation from a glassy solid state to a liquid state as an autocatalytic reaction-diffusion process. The velocity *V*_f_ of reaction-diffusion fronts of this kind normally scales as *V*_f_ ∼ (*DK*)^1/2^, where *D* is the effective translational diffusion coefficient of the reacting species and *K* is a rate constant governing the conversion of material from the unstable state to a more stable state.^64,80^ In the present context, it seems plausible to estimate *K* by the collective diffusion coefficient *D*_c_, which governs the rate at which large-scale composition gradients regress in miscible liquids. The dissolution of the polymer films clearly represents a large-scale decay of compositional heterogeneity. Far from any critical point for phase separation, *D*_c_ is predicted from van Hove theory^81^ to scale as the osmotic compressibility times the mobility. If we ignore the temperature dependence of the osmotic compressibility factor, which describes the driving force for mixing in a first approximation, and assume that the mobility scales inversely with the viscosity (*η*) of the material, as is often done, then we should expect the front velocity to scale in the present system to scale as *V*_f_ ∼ (*D*/*η*)­1/2. This scaling relation has been observed as a good approximation in Case II dissolution by Lasky et al.,^4–6^ a trend attributed by these authors to Thomas and Windle.^2,3,45,46^ The alternative plausible assumption that the mobility scales with *D* implies that *V*_f_ ∼ *D*, a relationship consistent with observations on the melting of vapor-deposited glass films.^36^ Some authors, including Thomas and Windle^2,3,45,46^ who have made many contributions to the topic of Case II diffusion, have emphasized the thermodynamic driving force, quantified by the osmotic pressure, to the rate of mixing. This extension of the purely kinetic argument for *V*_f_ is discussed by Qian and Taylor.^82^ The osmotic compressibility of a mixture in the one-phase region is well-known to correlate with the low-angle scattering intensity, denoted as *S*(0). This intensity generally scales linearly with the temperature difference (*T* – *T*_crit_), where *T*_crit_ is the critical temperature for phase separation, indicating the onset of mixture immiscibility. This scaling is general in mean field theories of phase separation, but a more complicated temperature dependence arises from mode-coupling effects.^83^ Our polymers are apparently highly miscible in the studied temperature range, so that the mean field theory estimate of the temperature dependence of the osmotic compressibility should be a good approximation. We thus might expect *V*_f_ to scale as *V*_f_ ∼ (*T* – *T*_crit_)^1/2^, if the thermodynamic driving force dominates *V*_f_ over the kinetic factors associated with the mobility. Interestingly, our data seems to conform with a scaling relation of this kind to a reasonable approximation, suggesting an apparent characteristic temperature *T*_crit_ of 129 °C. This scaling seems to remind us that the rate of the polymer intermixing should slow as a natural consequence of the polymer films becoming thermodynamically immiscible, so that the phase-separated state is the thermodynamically stable state. However, the temperature mentioned is more of an operational fit rather than a definitive characteristic temperature, and its significance remains unclear. The DSC curves of blended samples (see SM Figure S1) suggest that the blend is likely miscible, showing no distinct separation of the components within the sensitivity limits of DSC. In fact, the phase behaviors of PC/PMMA blends have been proven to be complicated, due to the interplay between thermodynamic driving force and kinetic factors.^84–92^ We conclude that the observed temperature dependence of the front velocity is qualitatively in line with previous experimental studies of Case II diffusion dynamics, but further studies are required to better understand the dependence of the front velocity on temperature and other relevant factors influencing the kinetic and thermodynamic properties of the glassy materials. The reaction-diffusion model discussed in the present paper provides a promising framework for developing a more rational understanding of this type of mixing process.

## Conclusions

Many commonly encountered materials, such as synthetic polymer materials, foods, personal care products, drugs, are in a glassy state in the sense of being in a rheologically defined solid state below *T*_g_ or in a “viscous fluid” state above *T*_g_, in which relaxation and diffusion are highly non-Arrhenius and large deviations from simple diffusive or “Fickian” type transport are frequently observed. In the present work, we examine the practical ramifications of this phenomenon in relation to how two miscible glassy polymers mix to form a material of uniform average composition. We study the dynamics of this phenomenon in high resolution by spin-casting polymer films and stacking the films into a bilayer to enable the interfacial dynamics using NR under controlled annealing conditions in which the films are thermally miscible and around *T*_g_ so that there is sufficient molecular mobility for the films to mutually mix toward a uniform composition state, apart from some composition variations near the boundaries of the stacked films that arise due the polymer surface interactions in those regions. Our goal was to determine the impact of the non-Fickian dynamics on this mixing process, which can be expected to arise in diverse material systems in which polymers and other glassy materials are “dissolved” by solvents or other polymers with which they are soluble.

Our NR measurements show that the physics of glass-formation can greatly alter the dissolution dynamics of polymer materials from the expectations of Fickian diffusion models. The significantly altered dynamics persist well above *T*_g_ of the polymer films. Instead of the well-known Fickian interdiffusion, we observe a mixing process similar to the dissolution of a “solid” material. Specifically, we observe a transient relaxation of the interfacial composition in the interfacial region between the polymer films to a composition comparable to the uniformly mixed material. The interfacial dynamics in this first stage of mutual film mixing somewhat resembled Fickian interdiffusion, although the width of the interfacial region coarsened with an apparent fractional power-law growth exponent near 1/3, as commonly observed in the coarsening of phase-separating polymer blends, rather than the Fickian value of 1/2. This non-Fickian scaling also aligns with observations in interfacial roughening in diverse pattern growth processes^64^ and tumor growth.^65^ These results highlight the complex nature of interfacial dynamics, with nontrivial exponents suggesting the influence of fluctuation effects that are often overlooked in conventional mean field models.

After an induction time *t* ^*^ over which the interfacial composition is relaxed locally, we observe a transition to a regime in which the “mixed polymer” domain between the films invades both films in a wave-like fashion. Phase field modeling of “solid” material dissolution was adopted as a framework for qualitatively characterizing the non-Fickian dissolution process up to a hypothesized characteristic temperature *T*_c_ (≈ 1.2 *T*_g_) in which a transition to a fluid-like state exhibiting Fickian dynamics was supposed to occur. The rates of the propagating composition wave, as well as the evolution of the interfacial width separating the relatively stable “uniformly mixed” polymer material from the relatively unstable unmixed polymer material, are quantified based on our NR data. The frontal dissolution process in glassy miscible polymer films was found to closely resemble the phenomenology of Case II non-Fickian diffusion commonly observed in the dissolution of glassy polymers by solvents. In this type of system, one of the layer components is not glassy, so that the dissolution wave propagates only in one direction. We thus suggest that Case II diffusion is a special case of the phenomenon that we observe in our stacked polymer films.

The coalescence of polymer materials in the form of polymer films and particles, encountered in 3-D printing, polymer recycling and other applications to name a few, and dissolution of polymer and other glassy materials (e.g., pharmaceuticals), arise in a vast number of applications, yet models of the dissolution dynamics of glassy materials tend to have limited general applicability and theoretical foundation. Our work provides fundamental new insights into the physical nature of this kind of ubiquitous dissolution process and the role of glassy dynamics in relation to the thermodynamic conditions in which non-Fickian mixing should be observed. Our admittedly heuristic comparison of our NR measurements to phase field theory modeling of the dissolution of model “solid” materials provides a new conceptual framework for understanding the mutual mixing of miscible glassy polymers and the dissolution of polymers in their glass state by small molecule solvents, a phenomenon previously termed Case II diffusion. We anticipate that this combination of high-resolution measurements and modeling will provide a foundation for developing a fundamental modeling, or at least a more successful general phenomenological description of dissolution processes in glassy materials.

## Supplementary Material



## Notes

The Supporting Information includes both data and detailed descriptions for: 1. Differential scanning calorimetry (DSC) measurement; 2. DREAM fitting of NR data with uncertainty analysis; 3. Evolution of SLD profiles for bilayer annealed at 130 °C; 4. Evolution of SLD profiles for bilayer annealed at 135 °C; 5. Evolution of SLD profiles for bilayer annealed at 140 °C; 6. Evolution of SLD profiles for bilayer annealed at 145 °C ; 7. Assessment of the uniformly mixed composition; 8. Evolution of SLD profiles for an immiscible bilayer annealed at 145 °C; 9. Thickness change of PMMA layer and dPC layer with time; 10. Front velocities *V*_mix_, *V*_dPC_ and *V*_PMMA_ (PDF)
