# Supplementary material for: A Neutron Reflection Study of the Dissolution of Miscible Glassy Polymer Films over a Range of Temperature
Source: Macromolecules. 2025 Oct 14;58(20):11137–48. doi: 10.1021/acs.macromol.5c02222 (PMC12574211; doi:10.1021/acs.macromol.5c02222)
Supplement: Supplementary file 1 [file ma5c02222_si_001.pdf]

Supplementary Materials:

A Neutron Reflection Study of the Dissolution of Miscible Glassy Polymer Films Over a Range of Temperature

Guangcui Yuan[1]*, *Sushil K. Satija*[1], *Thomas R. Murray*[2], *Jack F. Douglas*[3]*

[1]Center for Neutron Research, National Institute of Standards and Technology,

Gaithersburg, Maryland 20899, USA.

[2]Fayetteville State University, Department of Chemistry, Physics & Material Science, Fayetteville, NC 28301, USA

[3]Materials Science and Engineering Division, National Institute of Standards and Technology, Gaithersburg, MD 20899, USA

*Contact Author: guangcui.yuan@nist.gov

*Contact Author: jack.douglas@nist.gov

1. Differential scanning calorimetry (DSC) measurement

Various mixtures of dPC and PMMA were prepared by dissolving in a common solvent, methylene chloride, at a concentration of 5% by mass. The blend samples were poured into disposable aluminum pans which were placed in an oven set at 60 ˚C. These blends were subsequently dried at the same temperature under vacuum for a minimum of 24 h before further use. DSC was conducted using a Mettler Toledo DSC822e. The first scan was conducted up to 180 ˚C, followed by quenching the sample at a rate of 50 ˚C/min to 20 ˚C to initiate the second scan at a heating rate of 10 ˚C/min. The data presented here is from the second scan. All blends displayed very broad transitions instead of distinct transitions for each component. The DSC curves suggest that the blend is likely miscible, with no clear separation of the components within the sensitivity limits of the DSC. The broadening of the glass transition is associated with varying degrees of segmental mobility within the blend, where different polymer chains can influence one another's relaxation dynamics, resulting in a wider temperature range for the transition to take place.

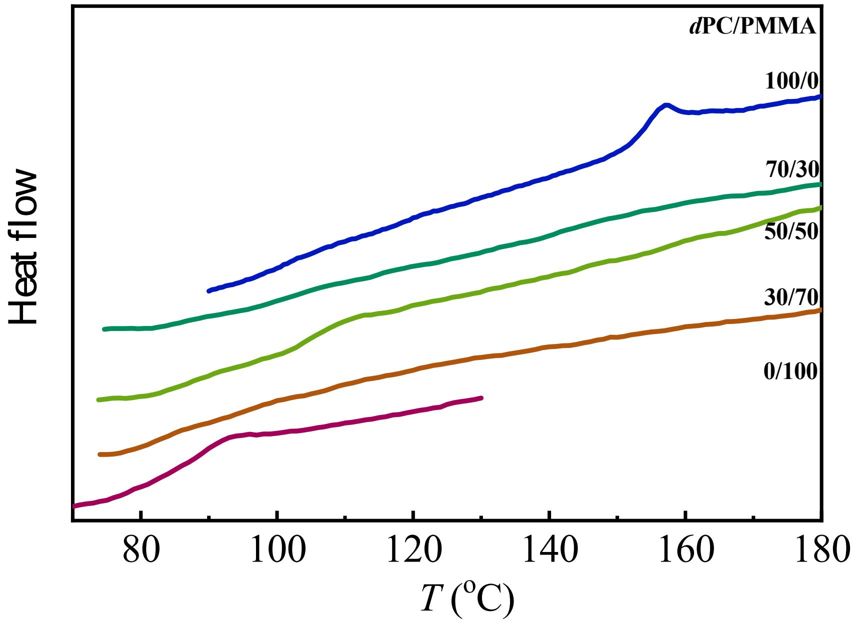

Figure S1. DSC traces of dPC and PMMA in different mixing ratios with a 10 °C/min heating rate.

2. DREAM fitting of NR data with uncertainty analysis

NR data was fit using Refl1D. Uncertainty in models and variables is usually a concern when interpreting the real space profile using the NR technique. The following is one example chosen to demonstrate the application of DREAM for model fitting and Bayesian uncertainty analysis in this study. The bilayer is annealed at 130 ^{o}C for 1320 mins, which is equivalent to 22 h. A certain extent of intermixing has occurred. The experimental reflectivity data are shown in **Figure S2(a)**. A stacked structure with five slabs is specified to fit the data: Si┃PMMA┃intermix┃dPC┃Air, as indicated by the gray vertical lines in **Figure S2(b)**. Each slab is described by three parameters: thickness (*l*), scattering length density (*SLD*), and interfacial width (σ, i.e., width of the interface with the next layer). The distance between two vertical lines corresponds to the thickness of the indicated slab. The thickness of medium air and substrate Si is assumed to be infinite, and their *SLD*s are constrained as 0 Å^{-2} and 2.07×10^{-6} Å^{-2}, respectively. Ten parameters listed in Table 1 are set as fit input for the DREAM algorithm to explore freely. The parameters of these slabs are allowed to vary over a wide range to account for possible compositional variations, thicknesses, and interfaces, with uniform probability in that range. The range is wide enough that some values may be out of the physically reasonable limits. For each parameter in the fit, DREAM finds the mean, median, and best values, as well as the 68 % and 95 % credible intervals. For this given example, the DREAM fit was completed with $\chi^2 = 5.436 \pm 0.081$, as shown in the solid line in **Figure S2(a)**. The corresponding *SLD* profile and composition profile are given in **Figure S2(b) and (c)**, respectively. The best values and the 95 % credible intervals are listed as the fit output in **Table S1**.

The uncertainty plot (**Figure S2(d)**) indicates that the fitting is sensitive to each parameter. Every parameter histogram in the uncertainty plot shows a narrow-distributed hump (instead of a box), indicating that the fit is sensitive to the corresponding parameter. The parameter histogram matches the green-constrained maximum likelihood line, indicating that the region around the best value has been adequately explored. The green asterisk represents the best value, the green *E* represents the mean value, and the vertical green line represents the median value. These three values are nearly coincident in the plot. Additionally, the correlation plot (**Figure S2(e)**) shows the cross-correlation between each pair of parameters. The nice blobs in the correlation plots indicate that the fit is sensitive to the corresponding parameters.

From the analysis, it can be concluded with great confidence that the model adopted in this study accurately represents the interfacial composition profile. Some information should be particularly mentioned. First and foremost are the SLD parameters in Table S1. Given a vast search space, the best-fit values of PMMA SLD and dPC SLD eventually fall at 1.06 × 10^{-6} $Å^{-2}$ and 3.30 × 10^{-6} $Å^{-2}$, which align well with the theoretical values for pure 100 % PMMA and 100 % dPC, respectively. Meanwhile, the SLD value of the intermixing layer, 2.28 × 10^{-6} $Å^{-2}$, is between two pure components, corresponding to a binary mixture with a dPC volume fraction of $\Phi_{dPC} = 0.54$. The SLD can be converted into a composition through

$$SLD = SLD_{PMMA}*\Phi_{PMMA} + SLD_{dPC}*\Phi_{dPC} \quad \text{(S1)}$$

in which volume fraction $\Phi_{PMMA} + \Phi_{dPC} =1$. The composition profile in **Figure S2(c)** is a corresponding conversion of the SLD profile using Eq. S1. In **Figure S2(c)**, we also demonstrate that the error function profiles can be closely approximated by a hyperbolic tangent (tanh) function using the corresponding slab parameters. The error function and the tanh function exhibit a high degree of similarity over this range. Therefore, taking into account experimental uncertainties, it is reasonable to conclude that the front profile can also be described using tanh profiles.

Table S1. Slab structure and fit parameters for a bilayer sample annealed at 130 °C.

Slab	Parameter	Fit input	Fit output dPC/PMMA
		Ranges	Best value [95% interval]
Si	σ_{Si} (Å)	0 - 20	9.88 [7.84 11.60]
PMMA	l_{PMMA} (Å)	0 - 500	369.8 [368.6 371.3]
	SLD_{PMMA} (×10^{-6} $Å^{-2}$)	0 - 2	1.06 [1.03 1.09]
	$\sigma_{PMMA\text{-}mix}$ (Å)	0 - 60	41.7 [39.4 43.1]
intermix	l_{mix} (Å)	0 - 400	235.0 [233.6 236.2]
	SLD_{mix} (×10^{-6} $Å^{-2}$)	1 – 4	2.31 [2.29 2.32]
	$\sigma_{mix\text{-}dPC}$(Å)	0 - 200	23.9 [22.3 24.7]
dPC	l_{dPC} (Å)	0 - 700	524.5 [523.8 525.3]
	SLD_{dPC}(×10^{-6} $Å^{-2}$)	2 - 4	3.30 [3.29 3.31]
	$\sigma_{dPC\text{-}air}$ (Å)	0 - 20	2.21 [1.61 2.62]
Air			

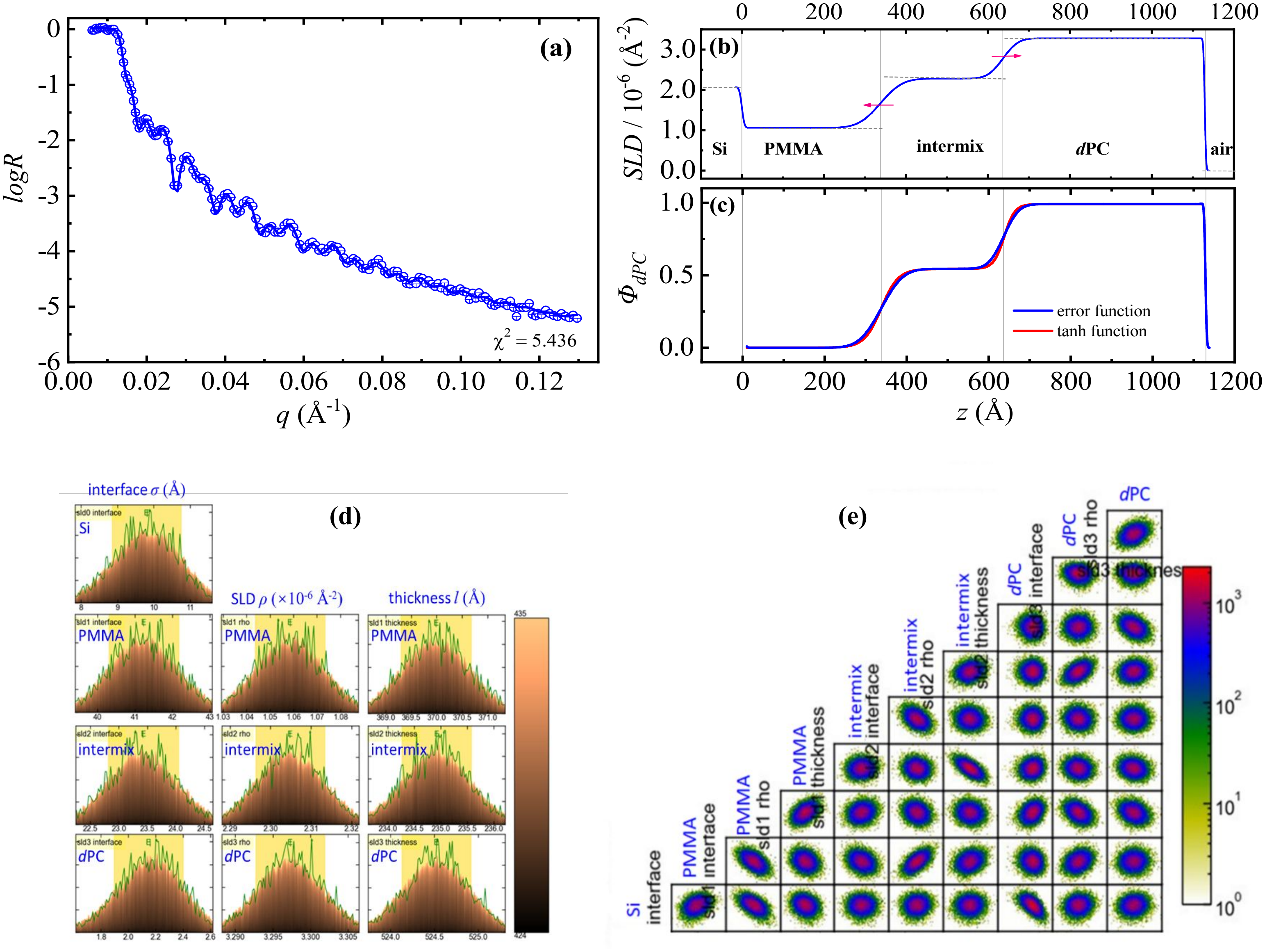

Figure S2 **(a)** Reflectivity curve. The symbol is the experimental data, and the solid line is the theoretical best fit. **(b)**The corresponding SLD profile. The vertical lines define the slabs. The horizontal dashed lines are references to the slab SLDs. The arrows point to the advancing directions of two fronts as annealing proceeded. (c) The corresponding composition profile according to the default error-function fit, and the comparison with the tanh-function profile with the same parameters. (d) Uncertainty plots of all fitted parameters. The histogram range represents the 95% credible interval, and the yellow-shaded region represents the 68% credible interval. (e) Correlation plots of all fitted parameters. The range plotted on the correlation plot is determined by the 95% interval of the data.

3. Evolution of SLD profiles for bilayer annealed at 130 ^{o}C

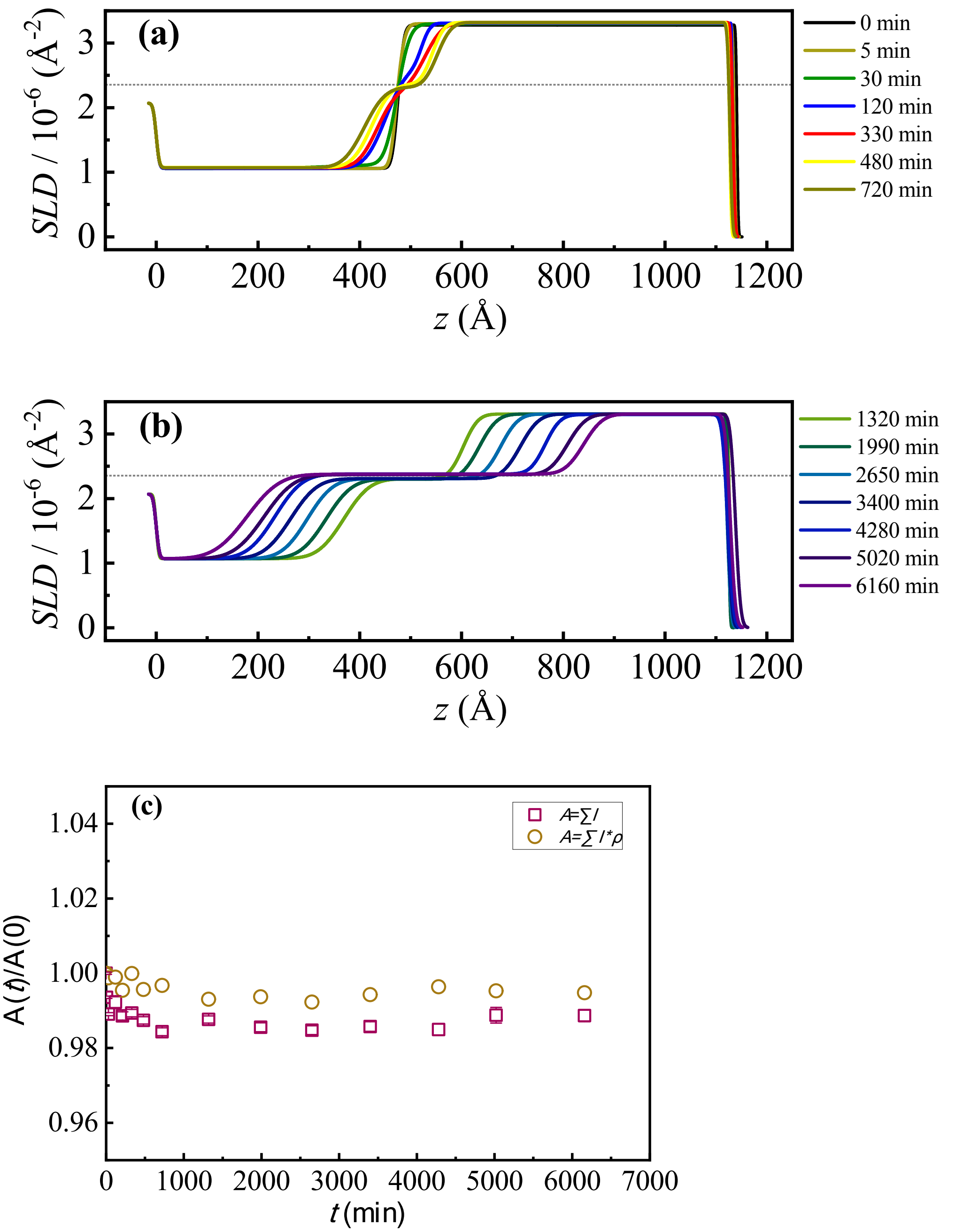

Figure S3. SLD profiles for a dPC/PMMA bilayer sample annealed at 130 ^{o}C (a) 0 -720 min "interfacial healing, (b) 1320-6160 min "frontal dissolution". The horizontal reference line indicates the theoretical SLD for a uniform mixture. (c) The relative variation of overall thickness and overall mass between all SLD fit profiles, compared to the as-cast film. Error bars show the 95% credible interval of the DREAM fit.

4. Evolution of SLD profiles for bilayer annealed at 135 °C

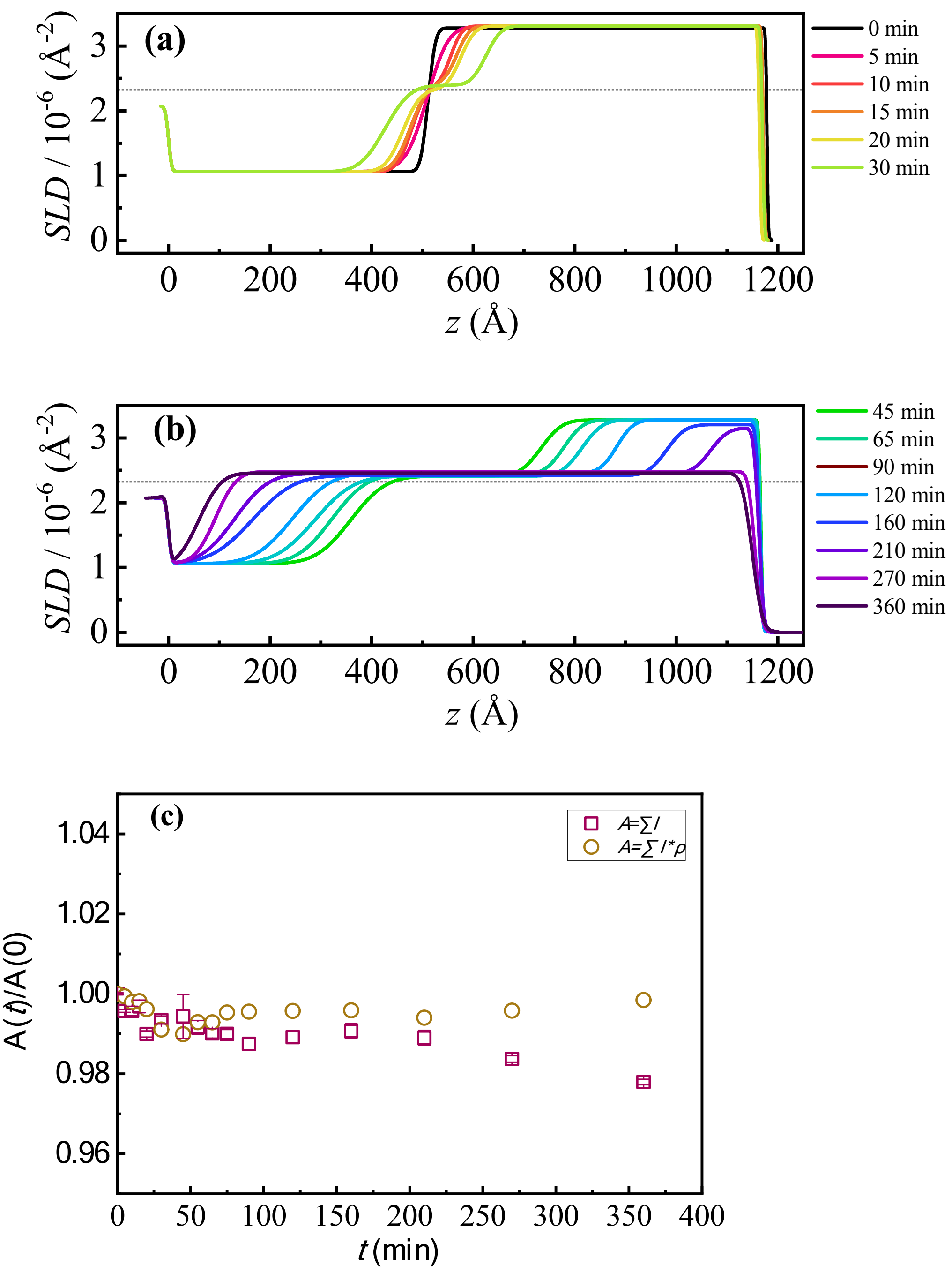

Figure S4. SLD profiles for a dPC/PMMA bilayer sample annealed at 135 °C (a) 0 -30 min, (b) 45-360 min. The horizontal reference line indicates the theoretical SLD for a uniform mixture. (c) The relative variation of overall thickness and overall mass between all SLD fit profiles, compared to the as-cast film. Error bars show the 95% credible interval of the DREAM fit.

5. Evolution of SLD profiles for bilayer annealed at 140 °C

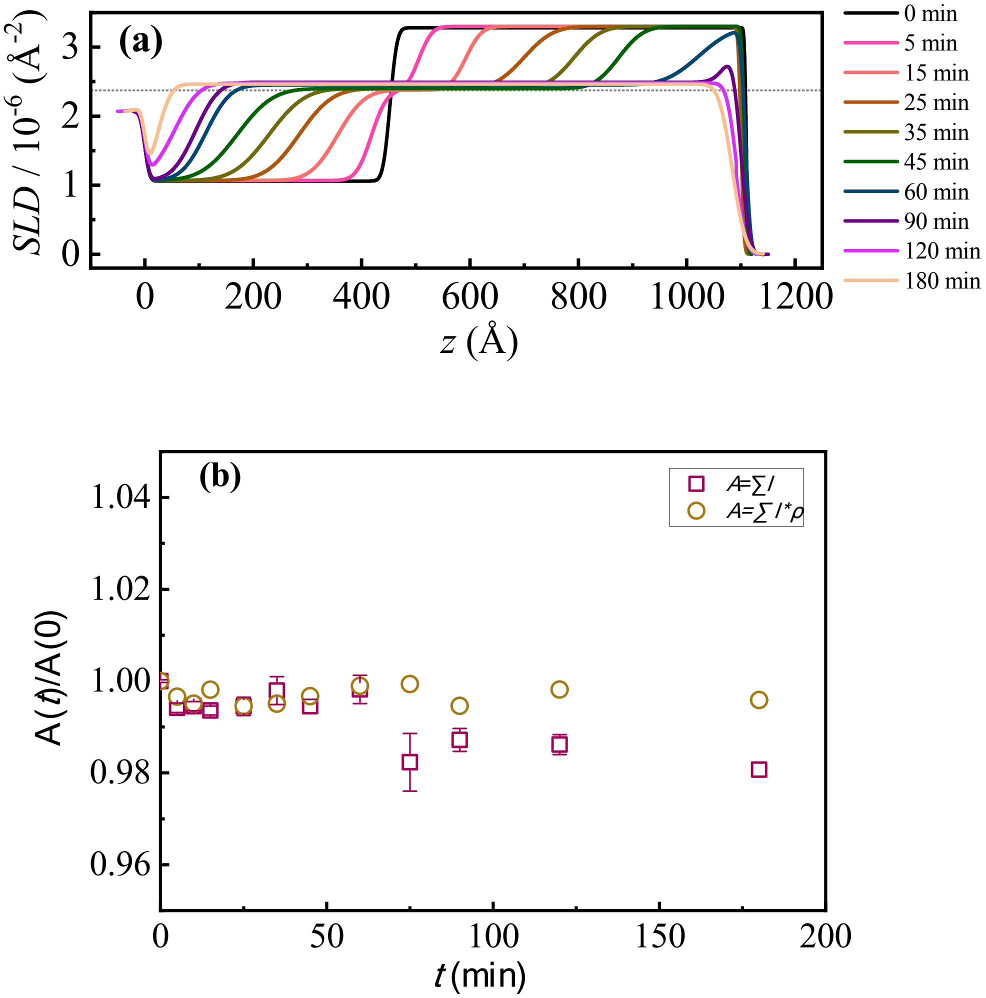

Figure S5. (a) SLD profiles for a dPC/PMMA bilayer sample annealed at 140 °C for 0 -180 min. The horizontal reference line indicates the theoretical SLD for a uniform mixture. (b) The relative variation of overall thickness and overall mass between all SLD fit profiles, compared to the as-cast film. Error bars show the 95% credible interval of the DREAM fit.

6. Evolution of SLD profiles for bilayer annealed at 145 °C

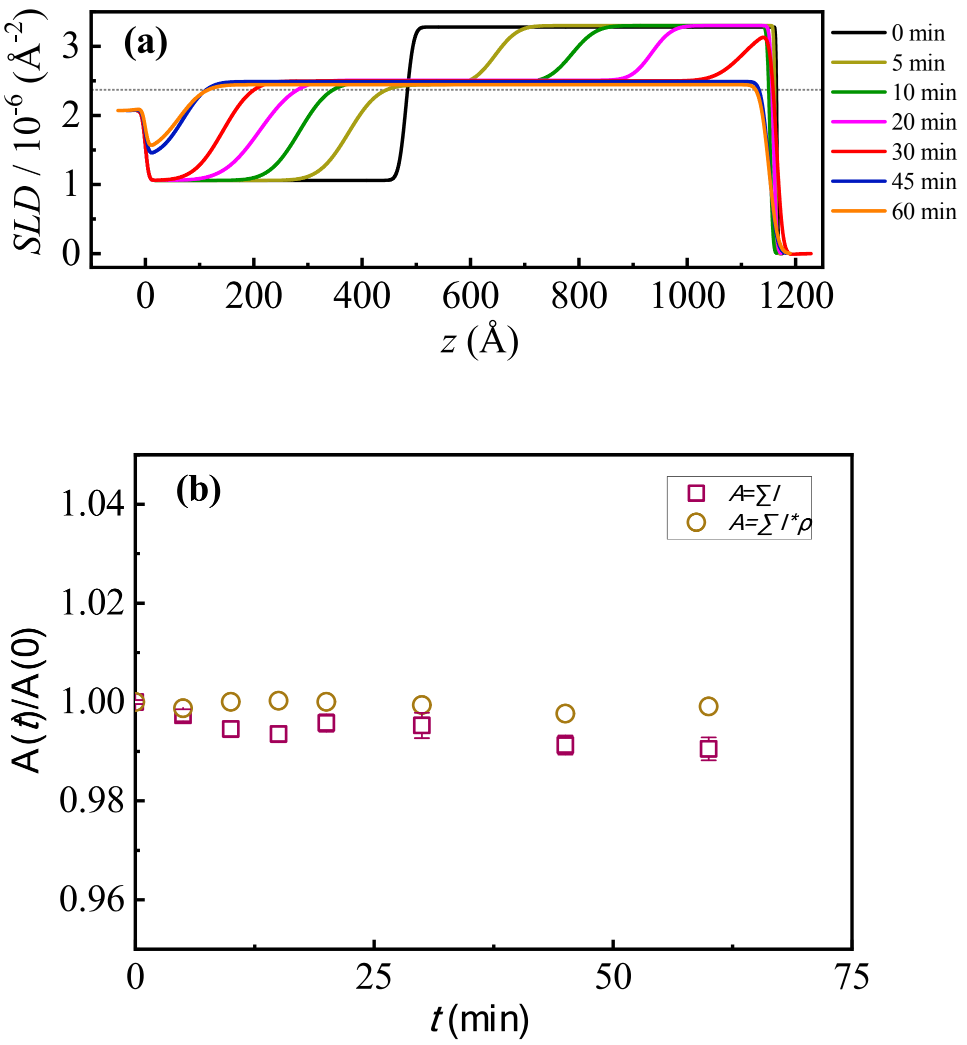

Figure S6. (a) SLD profiles for a dPC/PMMA bilayer sample annealed at 145 °C for 0 -60 min. The horizontal reference line indicates the theoretical SLD for a uniform mixture. (b) The relative variation of overall thickness and overall mass between all SLD fit profiles, compared to the as-cast film. Error bars show the 95% credible interval of the DREAM fit.

7. Assessment of the uniformly mixed composition

As we have mentioned in the main text, there is an induction period in which the interfacial region between two pure polymer films evolves to an intermediate layer with a composition that is apparently comparable to Φ_∞. After the induction time for this compositionally relaxed interfacial region to develop, the thickness of this intermediate mixed region increases as the propagating fronts extend into both pure films, where the rate of each front differs. In this frontal region, the composition transitions from that of the pure films to a mixed state with composition close to Φ_∞. **Figure 6S** compares the average of the observed composition Φ and the theoretical Φ_∞. Φ_∞ is slightly different for each film as it is given by the film thickness during preparation. The deviation of Φ at all four temperatures is less than ± 5% of Φ_∞, as indicated by the shape region. The slight deviation is inevitable, considering the following factors. First, the impact of diffusive interfaces has been overlooked in the calculation of Φ_∞. Second, the preferential affinity of the polymer components for the boundaries of the films leads to compositional heterogeneity very near the boundaries in the long-time limit.

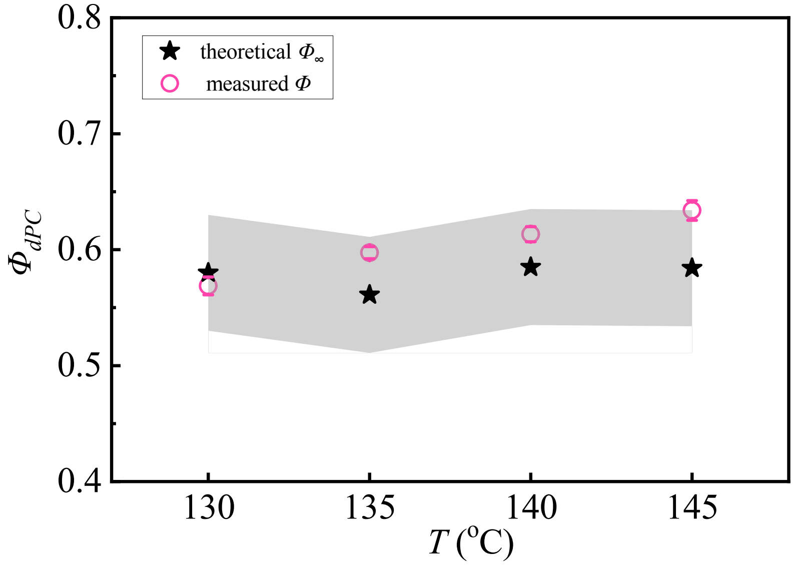

Figure S7: Comparison of the measured Φ and the theoretically calculated Φ_∞ at four temperatures. The error bars represent the standard deviation of Φ averaged over different annealing times. The shape region indicates ± 5 % of Φ_∞.

8. Evolution of SLD profiles for an immiscible bilayer annealed at 145 °C

The data presented above pertains to miscible polymers. To emphasize the differences with immiscible systems, we present the evolution of SLD profiles in a bilayer of dPC and high molecular weight PMMA (M_n = 222000 g/mol). Due to the increased molecular weight of PMMA, these two polymers are immiscible; however, some mobility is still observed in the films within the tested temperature range. The system does not maintain a uniform composition as it evolves into coexisting phases. This specific aspect will be discussed in more detail in a separate paper.

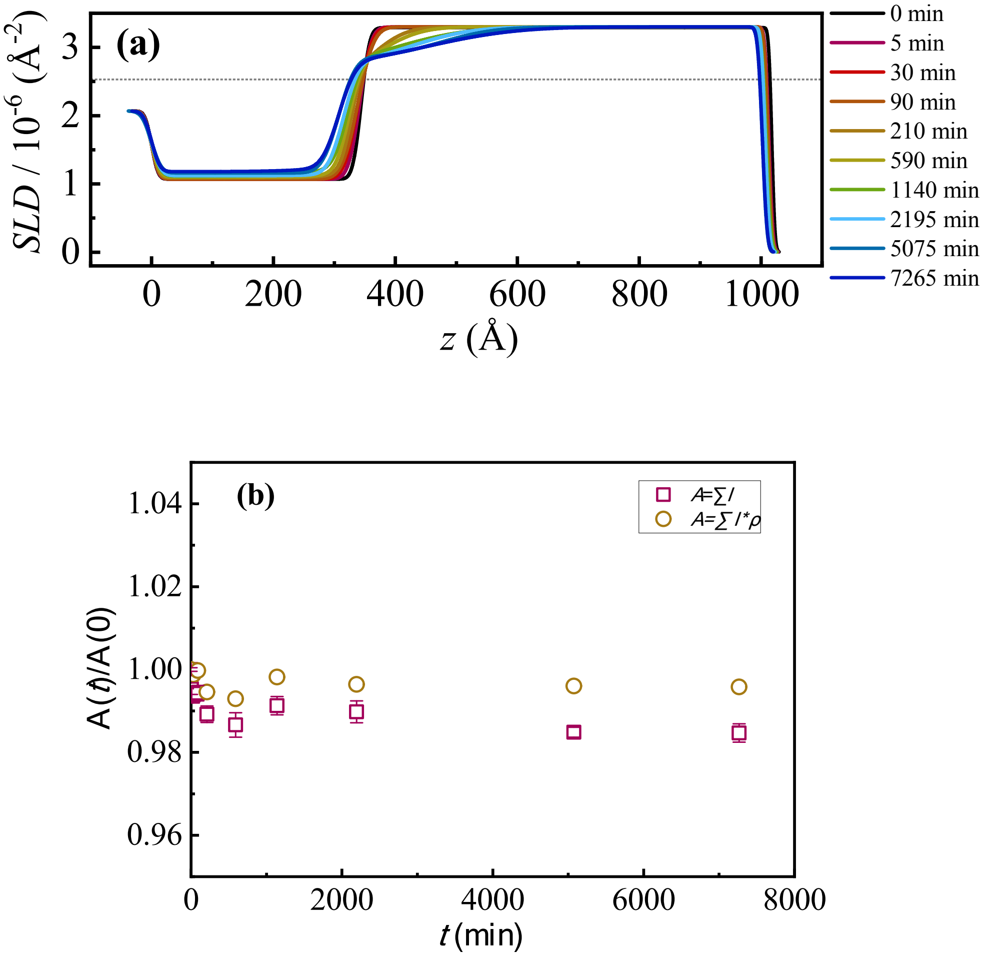

Figure S8. (a) SLD profiles for an immiscible dPC/PMMA bilayer sample annealed at 145 °C for 0 -7265 min. The horizontal reference line indicates the theoretical SLD for a uniform mixture. (b) The relative variation of overall thickness and overall mass between all SLD fit profiles, compared to the as-cast film. Error bars show the 95% credible interval of DREAM fit.

9. Thickness change of PMMA layer and dPC layer with time

l_{dPC} refers to the thickness of the dPC layer, and $\Delta l_{dPC} = l_{dPC}(0) - l_{dPC}(t)$ denotes the thickness decrease of the pure dPC layer as the mixing proceeds after time t. Here, $l_{dPC}(0)$ equals the as-cast thickness of dPC. Meanwhile, l_{PMMA} refers to the thickness of the PMMA layer and $\Delta l_{PMMA} = l_{PMMA}(0) - l_{PMMA}(t)$ denotes the thickness decrease of the pure PMMA layer as the mixing proceeds after time t. Here, $l_{PMMA}(0)$ equals the as-cast thickness of PMMA.

(a)

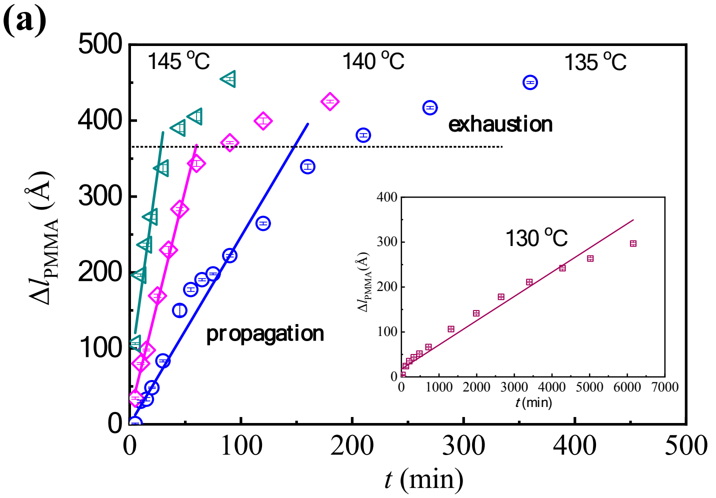

(b)

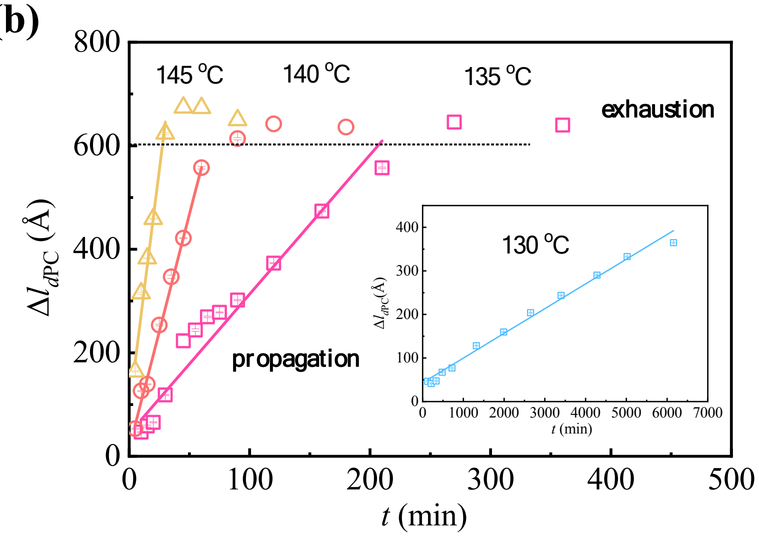

Figure S9. Thickness reduction with regard to the pure PMMA layer (a) and the pure dPC layer (b) as a function of annealing time (t) for bilayer samples annealing at 130 °C, 135 °C, 140 °C and 145 °C. Solid lines correspond to the linear fit of the propagation stage. Error bars show the 95% credible interval of DREAM fit.

10. Front velocities V_{mix}, V_{dPC} and V_{PMMA}

Table S2. The rate of thickness change in PMMA, intermix, and dPC layers during the propagation stage while annealed at 4 different temperatures.

T (°C)	V_{mix} (Å/min)	V_{PMMA} (Å/min)	V_{dPC} (Å/min)	$V_{mix}/(V_{PMMA}+V_{dPC})$	V_{dPC}/V_{PMMA}	$V_{PMMA}/(V_{PMMA}+V_{dPC})$
130	0.10 ± 0.001	0.054 ± 0.003	0.056 ± 0.001	0.91	1.04	0.49
135	5.46 ± 0.46	2.47 ± 0.14	2.69 ± 0.15	1.05	1.09	0.47
140	15.22 ± 0.65	5.92 ± 0.25	9.04 ± 0.33	1.02	1.53	0.39
145	26.14 ± 3.02	10.56 ± 1.73	18.50 ± 1.47	0.91	1.75	0.36

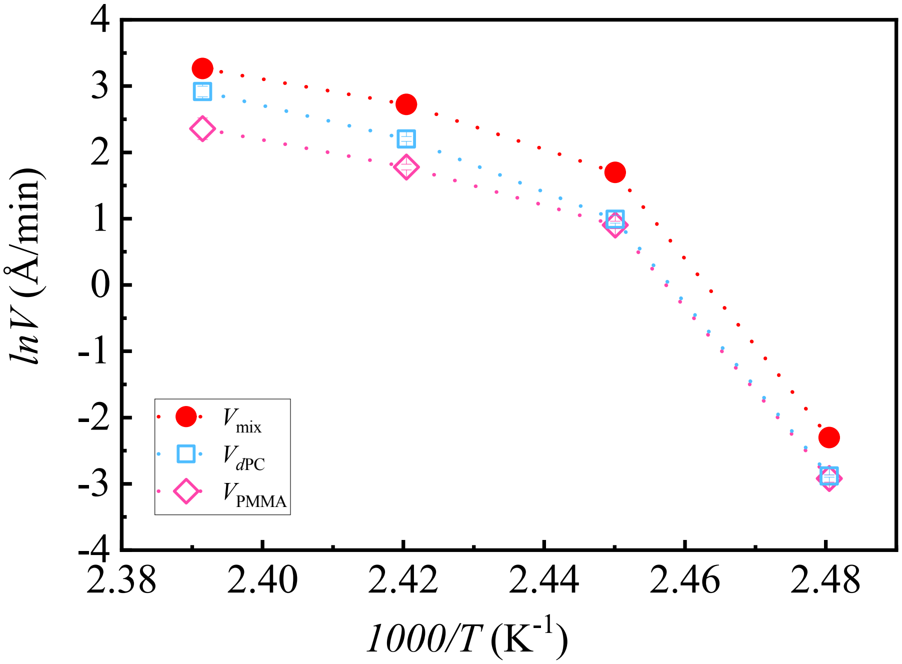

Figure S10. The relationship between V and T (in K) is shown in a ln$V$ vs. 1/T plot.
